# S1 nerve is the most efficient nerve rootlet innervating the anal canal and rectum in rats

**DOI:** 10.1038/srep13022

**Published:** 2015-08-11

**Authors:** Kai Fu, Pengbo Luo, Xianyou Zheng, Xiaozhong Zhu, Lei Wang, Yimin Chai

**Affiliations:** 1 Department of Orthopedic Surgery, Shanghai Jiaotong University Affiliated Sixth People’s Hospital, Shanghai 200233, China

## Abstract

Autonomic and somatic components participate in the defecation process in mammals, combining signals from the brainstem and forebrain. The innervation pattern involved in micturition in rats has been well studied, while defecation has been less studied. The aim of the present study was to identify the most important sensory and motor nerves of the anal canal and rectum involved in defecation. The amplitudes of evoked potential of the anal canal and rectum were higher when L6 and S1 ventral rootlets were stimulated, compared with the other segments (ANOVA and Tukey’s post hoc test, all *P* < 0.05). The S1 segment was more strongly cholera toxin subunit B conjugated to horseradish peroxidase (CB-HRP) positive compared with the other segments (ANOVA and Tukey’s post hoc test, *P* < 0.05). Ventral spinal rootlets of L6 and S1 mainly contributed to the pressure change in the anal canal and rectum when the ventral spinal rootlets from L5 to S3 were stimulated electrically. In conclusion, many afferent and efferent nerves innervate the anal canal and rectum and are involved in defecation, but the S1 nerve rootlet could be the most efficient one. These results could provide a basis for defecation reconstruction, especially for patients with spinal cord injuries.

Both autonomic and somatic components participate in defecation in mammals including sympathetic nerves from the caudal thoracic/upper lumbar cord, parasympathetic nerves from the sacral cord, and somatic motor nerves from the caudal lumbar/upper sacral cord^1^. However, inputs from both the brainstem and forebrain are necessary for the voluntary control of defecation^2^. Patients with spinal cord injury often suffer from impaired eliminative and sexual functions^1^,^3^. Studies have suggested that the rectal tone is increased after supraconal lesions and decreased after conal or cauda equina lesions^4^,^5^. Stimulation of the sacral parasympathetic nerves can increase the motility of the lower bowel and reduce constipation, which induce defecation in some patients in a manner analogous to post-stimulus voiding^6^.

The innervation pattern involved in micturition in rats has been well studied, while defecation has been less studied, and very few recent studies provide an accurate identification of the nerves involved in defecation in rats^7^,^8^,^9^,^10^. The most distal portion of the gastrointestinal tract is formed by the anal canal and the rectum, and both contribute to defecation. The aim of the present study was to identify the most important sensory and motor nerves of the anal canal and rectum involved in defecation.

## Results

### Nerve stimulation

**Figure 1:**
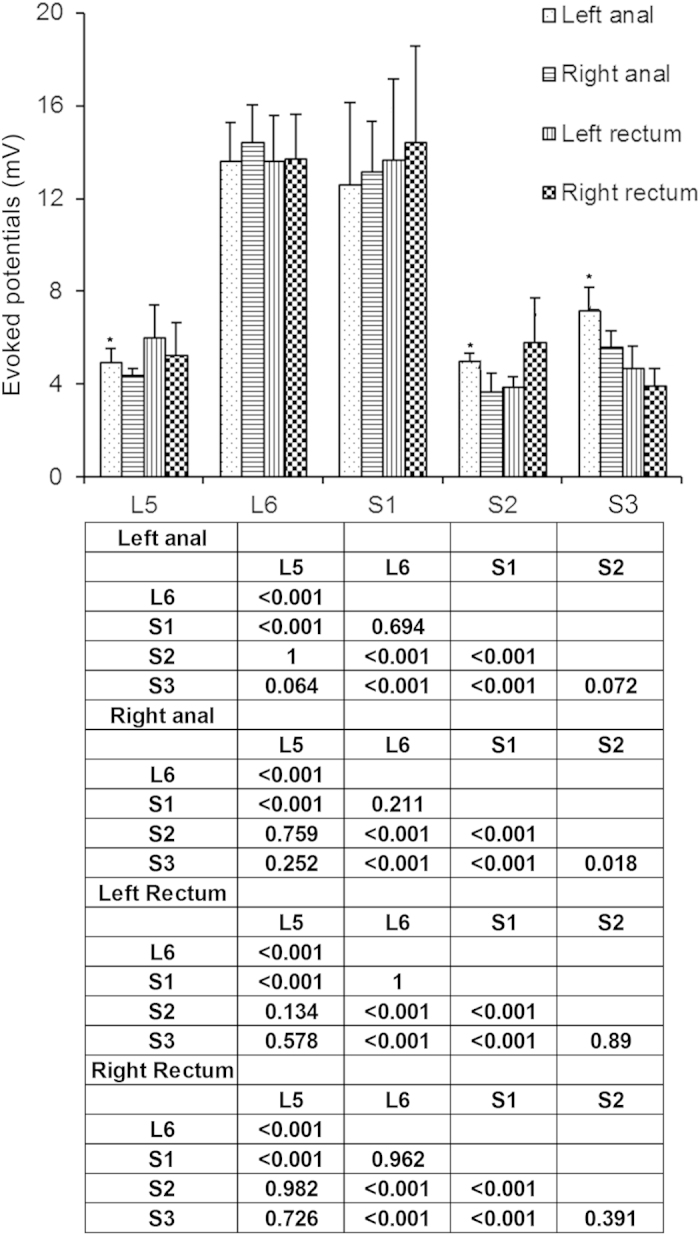
When single stimuli were performed to stimulate the spinal nerve rootlets from L5 to S3, bilateral amplitudes of evoked potentials (EP) of motor nerves innervating the anal canal and rectum were recorded. Data are shown as means ± standard deviation (SD) (n = 10). Statistical analysis was performed using ANOVA with the Tukey HSD test for post hoc analysis, or paired samples *t-*test, as appropriate. *P < 0.05 left *vs*. right (please see the Supplementary data). The matrix presents the *P*-values.

In order to characterize which nerves from the spinal cord segments innervate the rectum and theanal canal, the ventral nerve rootlets were sequentially stimulated using monophase square waves and the amplitude of the evoked potential was recorded (Supplementary Figure 1). The amplitude of evoked potential of the anal canal and rectum were higher when L6 and S1 ventral rootlets were stimulated, compared with the other segments (ANOVA and Tukey’s post hoc test, all *P* < 0.05) (Fig. 1 and Supplemental Table S1). Some differences were observed between the right and left rootlets in the anal canal (left and right L5, S2 and S3, ANOVA and Tukey’s post hoc test, *P* < 0.05), but not in the rectum (Fig. 1 and Supplemental Table S1).

### Cholera toxin retrograde tracing

**Figure 2:**
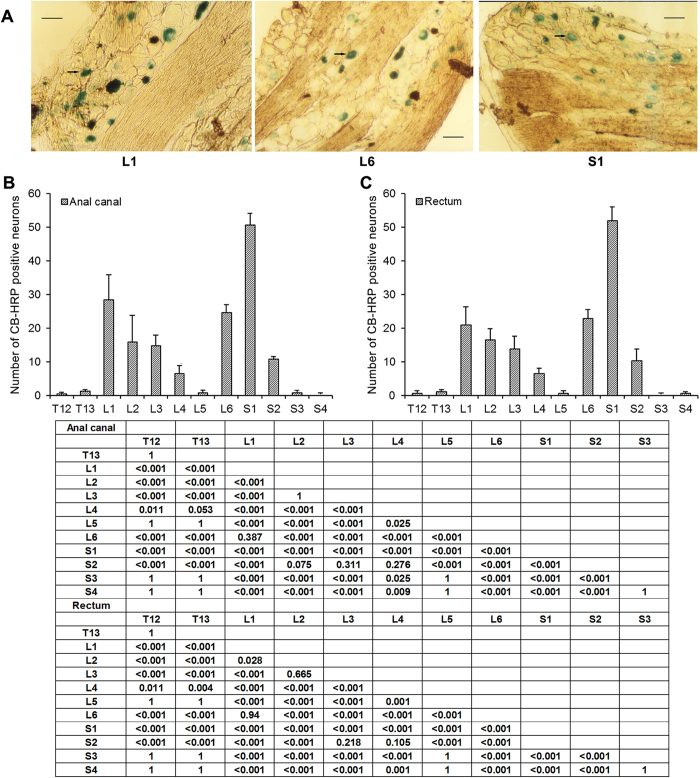
Sections taken through the dorsal root ganglia from rats by CB-HRP retrograde tracing showed positive staining in the cytoplasm (arrows). (**A**) CB-HRP-positive neurons in S1, L1 and L6 segments. Scale bar: 400 μm. (**B**) The most strongly CB-HRP-positive neurons were located in the S1 segment of the anal canal. (**C**) The most strongly CB-HRP-positive neurons were located in the S1 segment of the rectum. Data are shown as means ± standard deviation (SD) (n = 10). Statistical analysis was performed using ANOVA with the Tukey HSD test for post hoc analysis (please see the Supplementary data). The matrix presents the *P*-values.

To determine afferent fibers involved in the sensory pattern of the rectum and anal canal, cholera toxin subunit B conjugated to horseradish peroxidase (CB-HRP) was injected into the anal canal or rectum of rats, and neuron sections were examined. CB-HRP-positive neurons were found from T12 to S4 in the anal canal rectum (Fig. 2B,C, and Supplemental Table S2). Staining was observed in the cytoplasm, but not in the nuclei (Fig. 2A). The S1 segment was found as being the most strongly CB-HRP-positive compared with the other segments (ANOVA and Tukey’s post hoc test, all *P* < 0.05), followed by L1 and L6 (Fig. 2B,C, and Supplemental Table S2).

### Recording intraluminal pressure change

**Figure 3:**
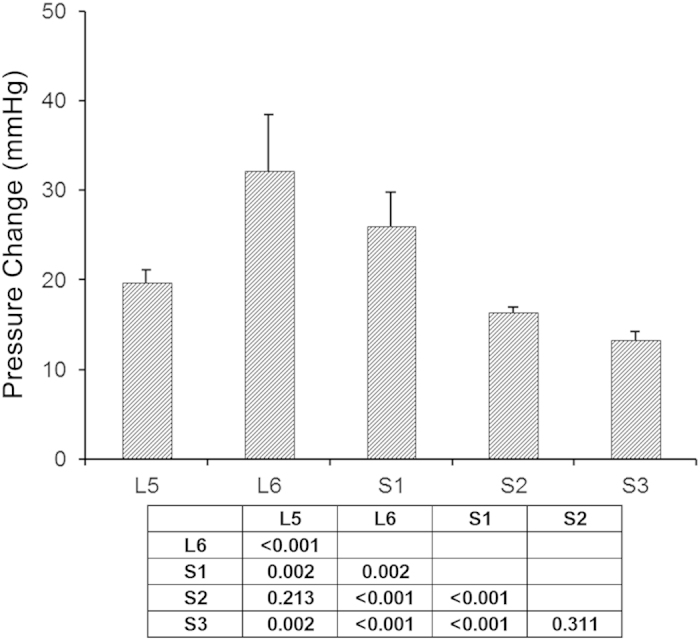
Intraluminal pressure was recorded from the anal canal and rectum in rats by a balloon-pressure transducer method. Intraluminal pressure was changed when each ventral spinal rootlet was stimulated. Ventral spinal rootlets of L6 and S1 mainly contributed to the pressure change. Data are shown as means ± SD (n = 10). Statistical analysis was performed using one-way ANOVA with the Tukey HSD test for post hoc analysis (please see the Supplementary data). The matrix presents the *P*-values.

In order to assess which nerves from the spinal cord segments contribute to the motor functions of the rectum and anal canal nerve roots were stimulated and the intraluminal pressure was recorded using a transducer. Propulsive contractions were produced by the rectum and anal canal when the ventral spinal rootlets from L5 to S3 were stimulated electrically. Although intraluminal pressure changed when each ventral spinal rootlet was stimulated, the pressure change was significantly different between different segments (ANOVA and Tukey’s post hoc test, all *P* < 0.05) (Fig. 3 and Supplemental Table S3). Ventral spinal rootlets of L6 and S1 mainly contributed to the pressure change (Fig. 3 and Supplemental Table S3).

## Discussion

The aim of the present study was to identify the most important sensory and motor nerves of the anal canal and rectum involved in defecation. The amplitudes of evoked potential of the anal canal and rectum were higher when L6 and S1 ventral rootlets were stimulated, compared with the other segments. Some differences were observed between left/right in L5, S2 and S3 in the anal canal, but not in the rectum. CB-HRP-positive neurons were found from T12 to S4 in the rectum. The S1 segment was found as being the most CB-HRP-positive compared with the other segments, followed by L1 and L6. Propulsive contractions were produced by the rectum and anal canal when the ventral spinal rootlets from L5 to S3 were stimulated electrically. Ventral spinal rootlets of L6 and S1 mainly contributed to the pressure change in the anal canal and rectum.

Neural control of the pelvic organs is a complex process involving both somatic and autonomic pathways participating in an exquisitely fine integration of segmental lumbo-sacral reflexes^11^. In humans, the normal process of defecation can be summarized as: 1) the distension of the rectum triggers the recto-anal inhibitory reflex and allows the feces to enter the rectum; 2) as filling continues, sensory information ascending to the brain leads to the sensation of rectal fullness; and 3) if the environment is appropriate for defecation, voluntary relaxation of the external sphincter occurs and peristalsis in the colon and rectum is initiated^12^,^13^,^14^. The intrinsic activity of the smooth muscles and the interaction with the enteric nervous system program the activity that is necessary to expel feces, but the external anal sphincter is controlled through somatic nerves with axons running in the pudendal nerves. The gut also receives input from the central nervous system through autonomic nerves, with parasympathetic input to the anorectum via the sacral roots (S2–S4) through the pelvic nerves, sympathetic input from the lumbar cord. However, voluntary effort to induce defecation can influence the control mechanisms^12^,^15^.

Some studies focused on defecation neurophysiology. Indeed, McKenna and Nadelhaft^16^ have shown that the pudendal nerve afferent neurons were located in the L6 and S1 dorsal root ganglia of Sprague-Dawley rats, and that the afferent neurons were larger and more numerous in males. Indeed, in the present study, similar evoked potentials were observed between L1 and S6. In addition, they also found that two motor neuron nuclei were labelled in the L5–L6 segments of the spinal cord. An experiment in rats showed that more pseudorabies virus-immunoreactive (PRV-IR) cells (identifying the neural control of colon function) were present in the region of the sacral parasympathetic nucleus (SPN) of the S1 spinal segment compared with that of the L6 segment^17^. According to the study by Morgan *et al.*^18^, a higher intensity distribution of afferent nerves within the SPN was observed in proximity to those neurons located in laminae V and VI, which innervate the colon, and a lower intensity near neurons located in Lamina VII, which innervate the bladder. This is consistent with the known spinal control of colon reflexes and the supraspinal control of bladder reflexes^18^. Luckensmeyer and Keast^19^ have demonstrated that the major pelvic ganglia provide their primary innervation of the intestine to the myenteric plexus, and are almost exclusively involved in the control of motility, and the pelvic innervation of the bowel is conducted via the rectal and penile nerves. Indeed, the plexuses in the rectum and distal colon are dense and irregular.

In the present study, the afferent neurons were distributed from the T12 to S4 segments in the dorsal horn of the spinal cord and dorsal root ganglion, mostly concentrated in the S1 segment. CB-HRP was used for neuron retrograde tracing. Indeed, CB-HRP is internalized by nerve fibers and undergoes retrograde transport to the neuron soma, which is not only fast and easy to implement, but also highly sensitive for pathway tracing studies of the nervous system^20^. However, an anterograde labeling to show the extent to which nerves project onto the rectal and anal muscles was hard to perform as the innervation of defecation is complex^1^. Innervation of the anal canal and rectum presents catholicity and centrality. Therefore, it may be believed that the S1 nerve rootlet is the most efficient nerve innervating sensory and motor function of anal canal and rectum in rats.

A skin-CNS-bladder pathway for restoring controllable reflex micturition was established by Xiao and Godec^21^. In this pathway, the impulses delivered from the efferent neurons of a somatic reflex arc can be transferred to initiate responses of an autonomic effector. However, this artificial reflex pathway is not intact, and triggers point defecation owing to the lack of awareness of defecation. In addition, high-level center of defecation cannot participate in the process, and defecation auxiliary muscles such as abdominal muscles and diaphragmatic muscles cannot be activated. As a result, reconstruction of motor and sensory pathways of defecation is paramount for patients with spinal cord injuries to recover the awareness of defecation.

The present study is not without its limitations. Indeed, it was performed in rats, and the results cannot be directly translated to humans. In addition, raw traces of the evoked potentials were not measured. We had to use the intraluminal pressure change method to study the defecation process instead of studying the physiological process, because rats had to be fasted for anesthesia, and because anesthetized rats often defecate involuntarily. Further studies are necessary before the results could be applied in a clinical trial.

In conclusion, many afferent and efferent nerves innervate the anal canal and rectum and are involved in defecation, but the S1 nerve rootlet could be the most efficient one. These results could provide a basis for defecation reconstruction, especially for patients with spinal cord injuries.

## Methods

The experiment protocol was approved by the Animal Experiment Ethics Committee of Shanghai Jiaotong University. The methods were carried out in accordance with the approved guidelines and regulations. Animals (Sprague-Dawley rats) used in this study were maintained in accordance with the Policy of Animal Care and Use Committee of Shanghai Jiaotong University.

### Animals

Fifty adult Sprague-Dawley rats were purchased from the Laboratory Animal Service Center of Shanghai Jiaotong University, and were housed at 20–25 °C and 50 ± 5% humidity with *ad libitum* access to food and water and 12:12 h light/dark cycle. All procedures and animal experiments were approved by the Animal Care and Use Committee of Shanghai Jiaotong University.

The rats (weighing 200–250 g at the time of experiment) were anesthetized with 1% pentobarbital sodium by intraperitoneal injection at 40 mg/kg. Penicillin was administered peri-operatively.

### Electrical stimulation using monophase square waves

Spinal rootlets from L5 to S3 were dissected under a Zeiss OPMI surgical microscope (Carl Zeiss, Oberkochen, Germany) (magnification: ×10). The receiving electrode of the Medtronic Keypoint two-channel electromyogram device was placed under the anal sphincter wall of the rats (Group A, n = 10). Stimulation using a monophase square wave was performed using an intensity of 2 mA, pulse duration of 0.2 ms, stimulation frequency of 5 Hz and persistent period of 5 s. The amplitudes of evoked potential of the motor nerves innervating the anal canal were recorded.

A median incision of the hypogastrium was made in rats (Group B, n = 10). The pelvic splanchnic nerves and the rectal plexus were exposed using a Zeiss OPMI surgical microscope (magnification: ×10). The receiving electrode was put into the rectum wall, and care was taken not to pierce the rectum. The amplitudes of evoked potential of the motor nerves innervating the rectum were recorded.

### Neural tracing using cholera toxin

With the rats (Group C, n = 10) lying supine under anesthesia, a median incision of the hypogastrium was made to expose the anal canal. CB-HRP (10 to 15 μl, 3 μg/μl) (Sigma, St Louise, MO, USA) was slowly injected into the anal canal wall using a micro-syringe into three sites equally distant from one to another. The needle was retained for 15 min and medical adhesive was applied to seal off the needle holes. The rats were kept alive for 48 h. Then, the rats were perfused transcardially with phosphate buffered saline (PBS), followed by buffered 4% paraformaldehyde. Spinal cords and bilateral dorsal root ganglia from T12 to S4 were removed and placed in PBS containing 20% sucrose at 4 °C for 24 hours. Forty-μm sections were obtained using a cryostat-microtome. HRP-positive neurons were shown using tetramethylbenzidine-sodium tungstate (TMB-ST) and observed using an Olympus BH-2 microscope equipped with brightfield and darkfield optics (Olympus, Tokyo, Japan). The proportions of CB-HRP-positive neurons in different segmental dorsal root ganglia were calculated using the Leica FW4000 image analysis system using one slide per animal.

CB-HRP positive neurons and afferent fibers of rat rectum (Group D, n = 10) from different spinal segments were analyzed using the same methodology as for the anal canal.

### Intraluminal pressure change

Intraluminal pressure change was recorded from the anal canal and rectum in rats (Group E, n = 10) using a balloon-pressure transducer method, as previously described^22^,^23^. The balloons were made from the head of rubber gloves and placed on the end of a polyethylene tube connected to a BL-410 transducer (ChengDu TME Technology Co., Ltd., ChengDu, China). The spinal rootlets for single stimuli from L5 to S3 were dissected under microscope. Stimulation intensity of 2.5 V, pulse duration of 0.2 ms, stimulation frequency of 5 Hz and persistent period of 4 s were used for stimuli of ventral spinal rootlets. The balloon was inserted through the anus to the anal canal and rectum, and kept in place during the whole procedure. Because of the fluctuations due to systolic and diastolic blood pressure, the baseline pressure was set as 50 mmHg^23^. Before electrical stimulation, the balloon was filled with 0.5 ml of normal saline. Then, single stimuli of the ventral spinal rootlets from L5 to S3 were performed, and the electrical stimulation was applied at 5 min intervals, during which the normal saline was given out to relax the anal canal and rectum.

### Statistical analysis

Results are presented as mean ± standard deviation (SD) and analyzed using one-way analysis of variance (ANOVA) with the Tukey HSD test for post hoc analysis, or the paired samples *t-*test, as appropriate. Statistical analysis was performed using SPSS 17.0 (SPSS, Chicago, IL, USA). Two-tailed *P*-values < 0.05 were considered statistically significant.

## Additional Information

**How to cite this article**: Fu, K. *et al.* S1 nerve is the most efficient nerve rootlet innervating the anal canal and rectum in rats. *Sci. Rep.* **5**, 13022; doi: 10.1038/srep13022 (2015).

## Supplementary Material

Supplementary data
