# Supplementary material for: S1 nerve is the most efficient nerve rootlet innervating the anal canal and rectum in rats
Source: Sci Rep. 2015 Aug 11;5:13022. doi: 10.1038/srep13022 (PMC4531329; doi:10.1038/srep13022)
Supplement: Supplementary data [file srep13022-s1.doc]

**S1 nerve is the most efficient nerve rootlet innervating the anal canal and rectum in rats**

Kai Fu, Pengbo Luo, Xianyou Zheng*, Xiaozhong Zhu, Lei Wang, Yimin Chai

Department of Orthopedic Surgery, Shanghai Jiaotong University Affiliated Sixth People’s Hospital, Shanghai 200233, China

Kai Fu and Pengbo Luo contributed equally to this work.

***Corresponding author: Xianyou Zheng** E-mail address:  [zhengxianyou@126.com](mailto:zhengxianyou@126.com)

Telephone number: +86-13916180968

Fax number: +86-2164701361

**Supplemental Table S1.** Bilateral amplitudes of evoked potentials (mV) in the anal canal and rectum

| Segments | Evoked potentials (mV) | | | |
| --- | --- | --- | --- | --- |
|  | Anal canal | | Rectum | |
|  | Left | Right | Left | Right |
| L5 | 4.93 ± 0.59^&^ | 4.37 ± 0.32 | 5.97 ± 1.46 | 5.23 ± 1.44 |
| L6 | 13.63 ± 1.64^***^ | 14.43 ± 1.62^***^ | 13.60 ± 1.96^***^ | 13.70 ± 1.94^***^ |
| S1 | 12.57 ± 3.55^***^ | 13.17 ± 2.14^***, #^ | 13.67 ± 3.47^***^ | 14.40 ± 4.15^***^ |
| S2 | 4.97 ± 0.37^###,ΔΔΔ, &&^ | 3.68 ± 0.77^###,ΔΔΔ^ | 3.87 ± 0.45^*,###,ΔΔΔ^ | 5.80 ± 1.91^###,ΔΔΔ^ |
| S3 | 7.17 ± 0.98^**,###,ΔΔΔ,▲,&&^ | 5.57 ± 0.74^*,###,ΔΔΔ,▲▲^ | 4.67 ± 0.98^###,ΔΔΔ^ | 3.93 ± 0.76^###,ΔΔΔ^ |

Data are shown as means ± standard deviation (SD) (n=10).

Statistical analysis was performed using one-way analysis of variance (ANOVA) with the Tukey HSD test for post hoc analysis, or paired samples *t*-test, as appropriate.

**P*<0.05, ***P*<0.01, ****P*<0.001 *vs.* L5; ^#^*P*<0.05, ^###^*P*<0.01 *vs.* L6; ^ΔΔΔ^*P*<0.05 *vs.* S1; ^▲^*P*<0.05, ^▲▲^*P*<0.01 *vs.* S2; ^&^*P*<0.05, ^&&^*P*<0.01 left *vs*. right.

**Supplemental Table S2.** Proportions of CB-HRP positive neurons

| Segments | Number of CB-HRP positive neurons | |  | |
| --- | --- | --- | --- | --- |
|  | Anal canal | Rectum | | |
| T12 | 0.40 ± 0.52 | 0.60 ± 0.70 | |  |
| T13 | 1.20 ± 0.42 | 1.10 ± 0.57 | |  |
| L1 | 28.40 ± 7.46^aaa,bbb^ | 20.90 ± 5.45^aaa,bbb^ | |  |
| L2 | 15.80 ± 7.91^aaa,bbb,ccc^ | 16.40 ± 3.40^aaa,bbb,c^ | |  |
| L3 | 14.80 ± 3.08^aaa,bbb,ccc^ | 13.80 ± 3.77^aaa,bbb,ccc^ | |  |
| L4 | 6.50 ± 2.32^a,ccc,ddd,eee^ | 6.40 ± 1.71^aa,bb,ccc,ddd,eee^ | |  |
| L5 | 0.80 ± 0.79^ccc,ddd,eee,f^ | 0.60 ± 0.70^ccc,ddd,eee,ff^ | |  |
| L6 | 24.50 ± 2.42^aaa,bbb,ddd,eee,fff,ggg^ | 22.80 ± 2.78^aaa,bbb,ddd,eee,fff,ggg^ | |  |
| S1 | 50.70 ± 3.37^aaa,bbb,ccc,ddd,eee,fff,ggg,hhh^ | 51.90 ± 4.12^aaa,bbb,ccc,ddd,eee,fff,ggg,hhh^ | |  |
| S2 | 10.70 ± 0.82^aaa,bbb,ccc,ggg,hhh,iii^ | 10.30 ± 3.53^aaa,bbb,ccc,ddd,ggg,hhh,iii^ | |  |
| S3 | 0.80 ± 0.79^ccc,ddd,eee,f,hhh,iii,jjj^ | 0.30 ± 0.48^ccc,ddd,eee,fff,hhh,iii,jjj^ | |  |
| S4 | 0.30 ± 0.48^ccc,ddd,eee,ff,hhh,iii,jjj^ | 0.60 ± 0.52^ccc,ddd,eee,ff,hhh,iii,jjj^ | |  |

Data are shown as means ± SD (n=10).

Statistical analysis was performed using ANOVA with the Tukey HSD test for post hoc analysis, or the Student’s *t*-test, as appropriate.

^aaa^*P*<0.001 *vs.* T12; ^bb^*P*<0.01, ^bbb^*P*<0.001 *vs.* T13; ^c^*P*<0.05, ^cc^*P*<0.01, ^ccc^*P*<0.001 *vs.* L1; ^d^*P*<0.05, ^dd^*P*<0.01, ^ddd^*P*<0.001 *vs.* L2; ^ee^*P*<0.01, ^eee^*P*<0.001 *vs.* L3; ^f^*P*<0.05, ^ff^*P*<0.01, ^fff^*P*<0.001 *vs*. L4; ^ggg^*P*<0.001 *vs*. L5; ^hhh^*P*<0.001 *vs.* L6; ^iii^*P*<0.001 *vs.* S1; ^jjj^*P<*0.001 *vs*. S2.

**Supplemental Table S3.** Intraluminal pressure changes following ventral spinal rootlets stimulation

| Segments | Pressure Change (mmHg) |
| --- | --- |
| L5 | 19.59±1.50 |
| L6 | 32.08±6.36^***^ |
| S1 | 25.89±3.90^**,##^ |
| S2 | 16.26±0.73^###,ΔΔΔ^ |
| S3 | 13.27±0.92^**,###,ΔΔΔ^ |

Data are shown as means ± SD (n=10).

Statistical analysis was performed using ANOVA with the Tukey HSD test for post hoc analysis.

**P*<0.05, ****P*<0.001 *vs.* L5; ^###^*P*<0.001 *vs.* L6; ^ΔΔΔ^*P*<0.001 *vs.* S1.


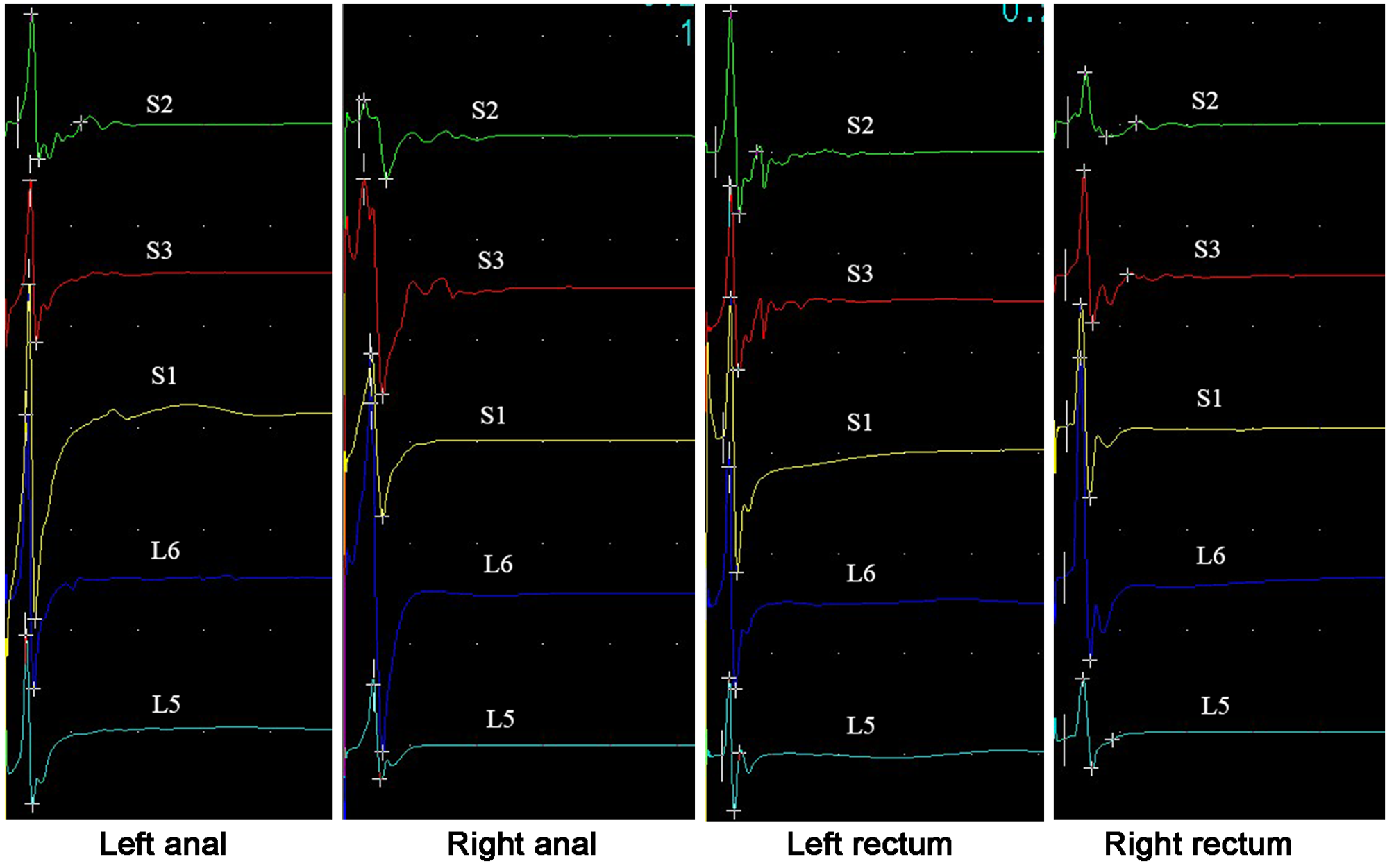


**Supplementary Figure 1.** Evoked potentials resulting from the sequential stimulation of ventral nerve rootlets using monophase square waves.
